# Global development of artificial intelligence in cancer field: a bibliometric analysis range from 1983 to 2022

**DOI:** 10.3389/fonc.2023.1215729

**Published:** 2023-07-14

**Authors:** Sui-Han Wang, Guoqiao Chen, Xin Zhong, Tianyu Lin, Yan Shen, Xiaoxiao Fan, Liping Cao

**Affiliations:** ^1^ Department of General Surgery, Sir Run Run Shaw Hospital, School of Medicine, Zhejiang University, Hangzhou, China; ^2^ Department of General Surgery, The First People’s Hospital of Yu Hang District, Hangzhou, China

**Keywords:** artificial intelligence, neoplasms, bibliometric analysis, deep learning, machine learning

## Abstract

**Background:**

Artificial intelligence (AI) is widely applied in cancer field nowadays. The aim of this study is to explore the hotspots and trends of AI in cancer research.

**Methods:**

The retrieval term includes four topic words (“tumor,” “cancer,” “carcinoma,” and “artificial intelligence”), which were searched in the database of Web of Science from January 1983 to December 2022. Then, we documented and processed all data, including the country, continent, Journal Impact Factor, and so on using the bibliometric software.

**Results:**

A total of 6,920 papers were collected and analyzed. We presented the annual publications and citations, most productive countries/regions, most influential scholars, the collaborations of journals and institutions, and research focus and hotspots in AI-based cancer research.

**Conclusion:**

This study systematically summarizes the current research overview of AI in cancer research so as to lay the foundation for future research.

## 1 Introduction

The concept of artificial intelligence (AI) was first proposed in 1956, which referred to the ability of machines to perform tasks that normally require human intelligence (1). As decades passed by, AI has already reshaped people’s life, especially in image recognition, speech recognition, and autonomous driving. Cancer remains one of the major challenges, and scientists turn to AI to conquer cancers in recent years. AI has shown great potential in various fields of cancer research and treatment. In the area of cancer diagnosis, it exhibits great diagnostic capacity, sometimes even comparable to human experts (2, 3). Currently, it is paving its path toward predict gene mutation models and charactering the tumor microenvironment (4, 5). The success of AI also extends to drugs design and efficacy predictions, which enormously decrease research costs and time burden (6, 7). As in patient prognosis and the response to therapy, the use of AI in this area gradually develops into a hotspot and attracts great attention (8).

Bibliometric analysis is a scientific method that uses statistical methods and mapping approaches to analyze patterns and trends in academic publications, which helps researchers quickly grasp research hotspots and trends in specific areas (9). In recent years, the publications of AI research in cancer have exponentially increased. It is of great significance to fully analyze and organize these literatures to present the research trends in this field. However, bibliometric analysis on the relationship between AI and cancer is still rare. Therefore, the aim of our study is to reveal the hotspots and trends of AI and tumor research so as to lay the foundation for future research.

In this study, we provided a global overview of tumor research in the field of AI from Web of Science (WOS). We performed a bibliometric analysis of publication and citation including annual growth, most productive countries/regions, most contributing authors, journals, and institutions. The worldwide co-authorship network and key contributors were analyzed by software. In addition, we identified the research focus and hotspots in AI-based tumor research.

## 2 Material and methods

### 2.1 Data extraction

The retrieval terms, namely, TS = tumor OR TS = cancer OR TS = carcinoma, AND AI, were searched in the Web of Science (WOS) core collection database from January 1983 to December 2022. All documents, including journal title, authors, abstract, published date, journal name, and so forth, were downloaded. The journal impact factor (IF) in the final records was obtained by manual processing in accordance with Journal Citation Reports (Clarivate Analytics, 2021).

### 2.2 Data collation

We documented and processed all data, namely, the country, continent, and Journal IF for each publication, using Microsoft Excel 2022. The country source was determined by the corresponding author’s address. For our study, we defined multiple countries cooperation (MCP) as any research with authors from different countries, and single country cooperation (SCP) as any research with author(s) from one country. The H-index, which is a quantitative metric that analyzes publication data by considering both the number of publications and their corresponding citations (10), of the author was obtained from the WOS database.

### 2.3 Statistical analysis

We analyzed the collated information using various visual analysis software tools. VOSviewer (version 1.6.19) and SCImago Graphica (version Beta 1.0.27) were used to visualize worldwide co-authorship. RStudio, based on R packages (biblimetrix, ggplot2, VennDiagram, and ggalluvial), was utilized to analyze publications from different countries, journals, and authors. To analyze keyword clusters, CiteSpace (version 6.1.R6) was used. Price’s Law (11) was used to select the number of core authors, and comprehensive index (Z index) (12) was calculated to identify chief pioneers based on author publication and citations. IF was considered to evaluate the quality of publications. For data analysis and correlation analysis, we used SPSS statistics software (version 26.0) and considered *P* < 0.05 as statistically significant.

## 3 Results

### 3.1 Global overview of tumor research in the field of artificial intelligence

**Figure 1:**
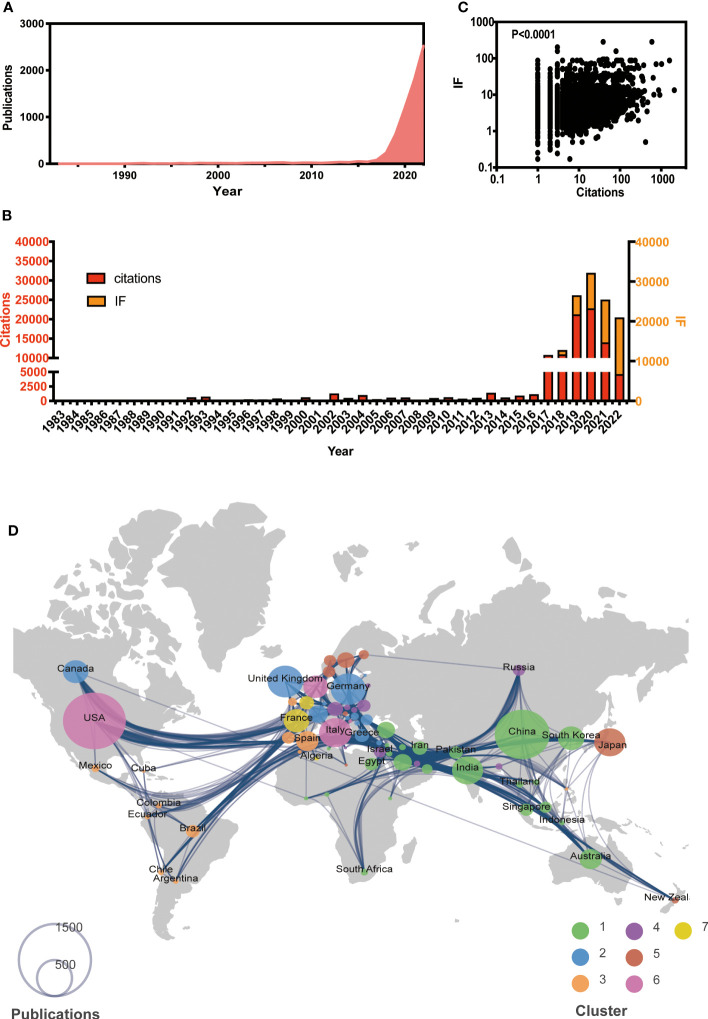
Global view of AI-based on tumor researches by the end of 2022. **(A)** Overall publication number over time. **(B)** Histogram of citation and impact factor (IF) over time. **(C)** Correlation analysis between citations and IF. **(D)** Global atlas of publications and country relationships. The colors on the map correspond to different clusters, while the size of the circle indicates the number of publications for each country (shown in the lower left corner). The lines between countries illustrate collaborations between them.

A total of 6,920 papers were collected, with the earliest paper dating back to 1983, as of the end of 2022. The number of publications has been gradually increased (**Figure 1A**). While the citations have shown an increasing trend until 2020 and have been decreasing thereafter, the journal IF has been steadily rising (**Figure 1B**). Furthermore, there is a significant correlation between IF and citations (*P* < 0.001, **Figure 1C**). Those suggest that AI-based tumor research has attracted much attention to improve the medical care.

Based on co-authorship, seven clusters of worldwide publications were identified (see **Figure 1D**). Cluster 1, led by China (*n* = 1,434), India (*n* = 457), and South Korea (*n* = 328), was primarily located in Asia. Cluster 2 included various European countries, such as the UK (*n* = 591), Germany (*n* = 578), and Greece (*n* = 99). Cluster 3 included Spain (*n* = 234) and several African countries. Notably, the USA had the largest number (*n* = 1,878) globally and was the leading country in Cluster 6. The leading country in Clusters 4, 5, and 7 was Austria (*n* = 117), Japan (*n* = 458), and France (*n* = 312), respectively.

### 3.2 The analysis and correlation of top countries and journals in AI-based tumor research

**Figure 2:**
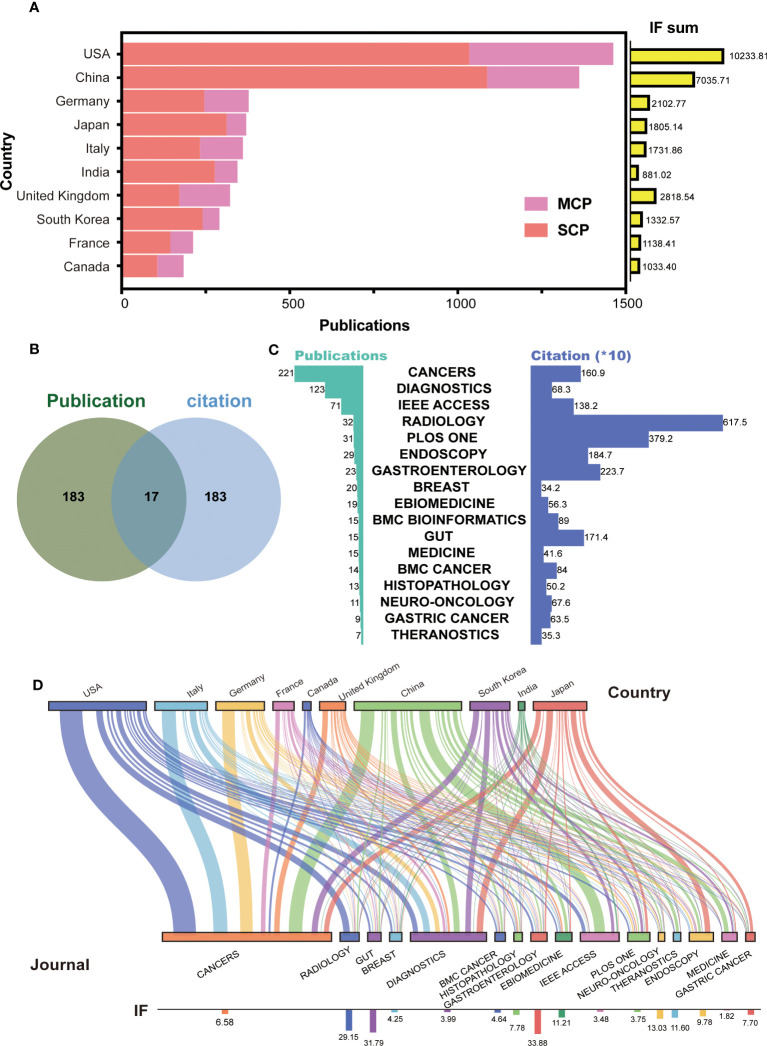
Top countries and journals in the field of AI-based on tumor researches. **(A)** The number of publications and IF in top 10 countries. MCP, multiple countries cooperation; SCP, single country cooperation; IF, impact factor. **(B)** Venn diagram between top 200 journal publication and top 200 journal citation. **(C)** The details for merged 17 journals with high publication and citation. **(D)** Sankey chart between top countries and journals.

To further identify the leading countries, the 6,920 papers were classified into SCP and MCP based on the corresponding authors’ addresses. The top 10 countries with the highest sum of publications and journal IF were selected and presented in **Figure 2A**. The results showed that the USA (*n* = 1,363, IF = 10,233.81) and China (*n* = 1,269, IF = 7,035.71) had highest number of publications and IF, with China having the highest number of SCP (*n* = 1,014). The publication numbers of other countries in the top 10 ranged from 174 to 354. Interestingly, Japan, South Korea, and India had the highest ratio of SCP to MCP, while the UK had the lowest ratio. Additionally, the IF sum of India (IF = 881.02) was lowest despite its high publication number, while the UK (IF = 2,818.54) ranked third in terms of journal IF. Those suggest that certain countries, such as the USA and China, have emerged as leaders in AI-based tumor research. On the other hand, some countries, such as Japan and South Korea, appear to conduct more independent research and development, while others, such as the UK, seem to collaborate more with other countries and publish higher quality papers.

In terms of the published journals, we initially selected the top 200 journals based on the number of publications and citations (**Supplementary Figures S1A, B**). Then, to identify the most excellent journals, we merged two clusters to obtain 17 journals (**Figure 2B**). These included *CANCERS*, *DIAGNOSTICS*, *IEEE ACCESS*, *RADIOLOGY*, *PLOS ONE*, *ENDOSCOPY*, *GASTROENTEROLOGY*, *BREAST*, *EBIOMEDICINE*, *BMC BIOINFORMATICS*, *GUT*, *MEDICINE, BMC CANCER*, *HISTOPATHOLOGY*, *NEURO-ONCOLOGY*, *GASTRIC CANCER*, and *THERANOSTICS*. *CANCERS* had the highest number of publications (*n* = 221), and *RADIOLOGY* had the highest number of citations (*n* = 6,175) (**Figure 2C**). The presentation of Sankey diagram further exhibited the tendency for journal in different countries (**Figure 2D**). As expected, *CANCERS* (IF = 6.58) and *DIAGNOSTICS* (IF = 3.99) gained popularity for most corresponding authors from different countries. American, British, and Japanese authors laid emphasis on the high-quality papers, and published on the journal of *RADIOLOGY* (IF = 29.15), *EBIOMEDICINE* (IF = 11.21), and *ENDOSCOPY* (IF = 9.78). Chinese authors published lots of papers on *IEEE ACCESS* (IF = 3.48). These results suggest that AI research based on tumors is seem to be more focused on tumor imaging and diagnosis.

### 3.3 The worldwide co-authorship network and key contributors

**Figure 3:**
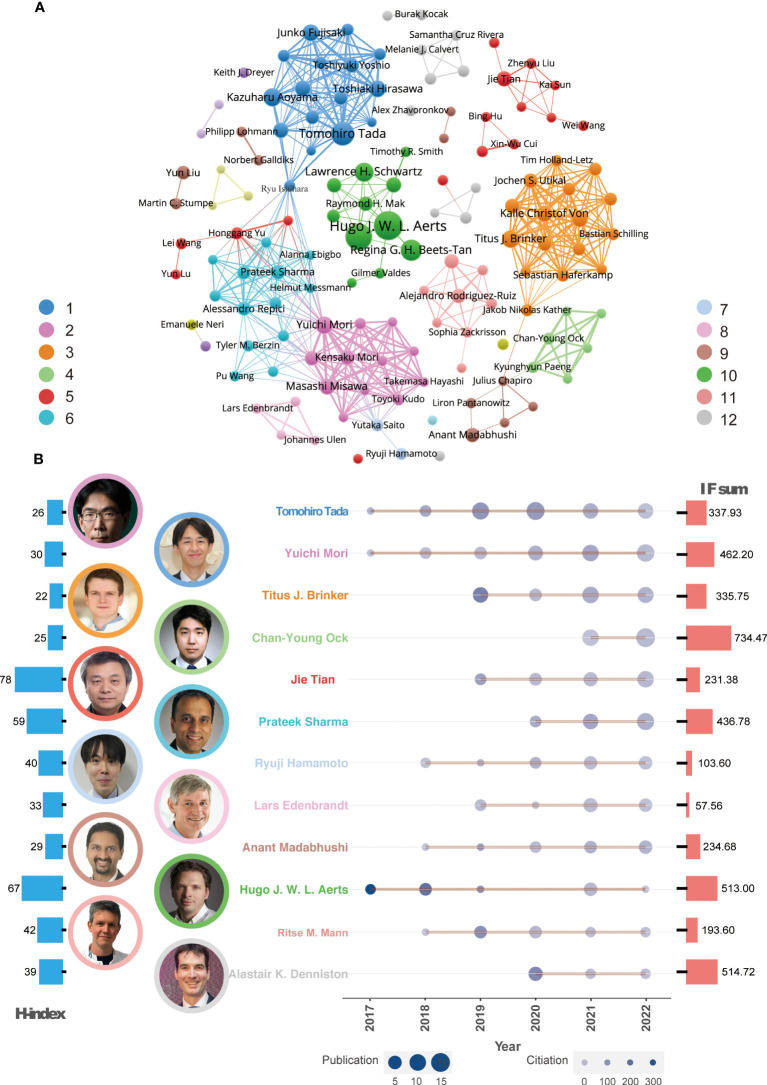
Global co-authorship network analysis and leading authors. **(A)** Co-authorship network and representative authors in each cluster. **(B)** Publication trends of the leading author in every cluster over time. The color of the authors’ names corresponds to the clusters in **Figure 2A**. The size of the circles in the line chart represents the number of publications, while the depth of the round color indicates the number of citations. The H-index and IF sum per author are depicted in the left (red) and right (blue) histograms of this panel, respectively.

To analyze the relationship between worldwide authors, we used VOSviewer software to create an author cooperation network. The core author groups were displayed and 12 clusters were identified (**Figure 3A**). Some clusters represented isolated teams, such as Clusters 10 and 5, while others were closely connected to each other through important intermediaries, such as Clusters 1 and 6 and Clusters 6 and 2.

According to the Z index for each author, 12 leading researchers were identified in each cluster (**Figure 3B**). Since 2017, many of these influential authors have been publishing AI-based tumor research papers and their publication output has gradually increased. These leading researchers include Professor Tada, Professor Mori, Professor Brinker, Professor Tian, Professor Sharma, Professor Hamamoto, and Professor Madabhushi. In terms of publication quality, Professor Ock, Professor Aerts, and Professor Denniston have published high-quality papers with a journal IF of more than 500. For evaluating the author’s influence, Professors Aerts and Tian were recognized as highly cited researchers based on their H-index.

### 3.4 Exploration of AI-based popular tumor research

**Figure 4:**
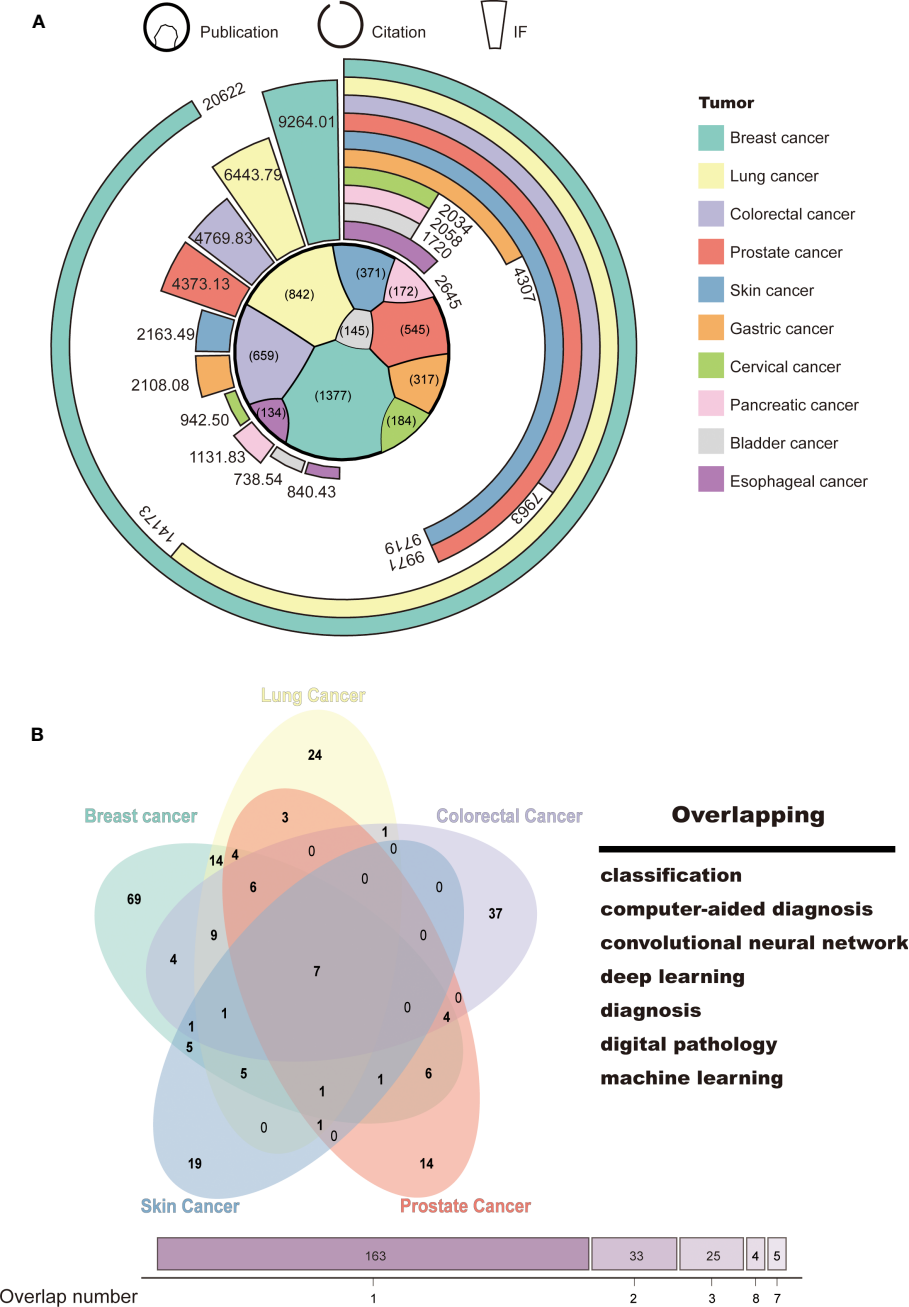
Tumor classification and hotspots for AI-based research. **(A)** Classification of tumor research. **(B)** Venn diagram of the top 5 tumors in terms of publications and research focus.

To identify the research focus and hotspots in AI-based tumor research, we conducted a frequency analysis of keywords using VOSviewer and CiteSpace bibliometric software. Keywords with high frequencies were clustered based on the developed techniques, such as “machine learning,” “convolutional neural network,” and “natural language processing,” and tumor research types, such as “breast cancer,” “lung cancer,” and “prostate cancer” (**Supplementary Figures S2A, B**). We then selected the top 10 tumor types according to the number of publications, namely, breast, lung, colorectal, prostate, skin, gastric, cervical, pancreatic, bladder, and esophageal cancer. More details on citation and journal IF were presented in **Figure 4A**. As expected, the ranking of citation and IF was roughly in line with the publication number. AI-based research on breast and lung cancer had both high-quantity and high-quality publications (Breast: publication: 1,377; citation: 20,622; IF: 9,264.01; Lung: publication: 842, citation: 14,173; IF: 6,443.79). This may be closely related to the global incidence of these types of cancer.

To explore the main functions of AI in tumor research, we collected and merged the keywords of the top 5 tumor types, which include breast, lung, colorectal, prostate, and skin cancer. Seven keywords were identified, namely, “classification,” “computer-aided diagnosis,” “convolutional neural network,” “deep learning,” “diagnosis,” “digital pathology,” and “machine learning” (**Figure 4B**). This suggests that AI-based tumor research currently focuses on classification and diagnosis through a variety of machine learning techniques.

## 4 Discussion

In the present study, we analyzed the main knowledge and trends of AI in cancer and found the publication number was soared from 2017. We considered this phenomenon mainly attribute to the following events. Esteva et al. published an influential article in Nature in 2017 (13). They found that the performance of the AI algorithms was nearly equal to that of the dermatologists in diagnosing skin cancer. In addition, the deep learning frameworks have made significant progress in 2017. Facebook’s PyTorch caused a huge sensation (14), Google’s Tensorflow developed rapidly in 2017 as well (15). The USA and China are the most productive countries. It is consistent with the current AI development trend and capabilities in the world. As shown in **Figure 1D**, international cooperation was very frequent, and countries with similar geography or language and culture cooperated more closely. At the government level, France and Canada established a working group to discuss the code of conduct in the use of AI, study the impact of introducing AI into the clinic, and collaborate on AI terminology, imaging data regulation, education, ethics, and future applications (16, 17). The majority of publications on *RADIOLOGY* belong to American authors, while the Japanese authors’ papers predominate in *ENDOSCOPY* and *GASTRIC CANCER*. The phenomena we noticed above show that American scholars focus mostly on medical imaging, while Japanese scholars’ research centers on endoscopy and gastric cancer. This may be related to the high incidence of gastric cancer and the high penetration of gastroscopy in Japan. In China, AI tumor research is also focused on medical imaging due to the ease of access to data. Professor Tada Tomohiro from Japan has published the largest number of the articles. His team mainly focus on the application of AI in endoscope (18–22). They successfully established AI systems to assist the endoscopic diagnosis of gastric and esophageal cancer. It is noteworthy that Professor Ock Chan-Young, who is the chief medical officer from a Korean AI company, achieved the highest total IF scores with his research papers among others, which implied medical AI is of huge commercial value (23). Professor Tian from China and Professor Alert from the USA who are both pioneers in the direction of AI Medical imaging received the highest H-index, indicating that their articles were the most cited. Professor Sharma’s team of the USA has participated in the most international collaborations, among which they work closely with Professors Mori’s and Tada’s teams of Japan, whose works mostly focus on the field of endoscopy. It is crucial to enhance the intensity of interdisciplinary collaborations in order to bridge different fields. Some authors play a significant role as connectors between computer science and the medical field. Professor Ishihara, with expertise in gastroenterology, has collaborated with Professor Sharma’s team to advance research on AI applications in gastroenterology cancer. Additionally, Professor Ishihara has actively collaborated with Professor Tada, resulting in numerous papers focused on the intersection of AI and gastroenterology since 2019. Professor Saito, affiliated with the National Cancer Center Hospital in Japan, serves as the director of the endoscopy center and chief of the endoscopy division. He has provided valuable gastroenterological data for computer-based deep learning and has fostered close collaborations with Professor Sharma’s computer science team. However, current interdisciplinary collaborations primarily focus on research between computer science and internal medicine physicians or radiologist. In the future, it is essential to pay attention to collaborations between computer science and surgeons, particularly in the context of tumor resection using robotic surgery (24).

“Machine learning” and “deep learning” are the most common keywords (**Supplementary Figure S2**). Deep learning, a subtype of machine learning, is based on convolutional neural networks (CNN) mainly addressing the fields of image, text, and speech, with issues focused on classification and regression (25). With the development of deep learning models, the application of AI in medicine has begun to make important progress. Most of the current AI research used in the field of tumor introduced deep learning algorithms. To improve the accuracy, researchers always mixed multiple algorithms together (26). Deep learning model can also be combined with multimodal data such as image and clinical data to improve accuracy (27, 28). Recently, transfer learning, another subset of machine learning, is to use a pre-trained model upon a dataset as a base point to solve a new related problem (29). Transfer learning, which is expected to lead the next wave of machine learning technology after deep learning, has also been used effectively for medical image analysis. Khan et al. fine-tuned a patch-based breast cancer lymph node dataset by using transfer learning to establish a computer-assisted diagnostic tool for lymph node screening in patients with colorectal cancer (30). We believe that, with the advent of new machine learning methods, AI will bring a revolution to the field of cancer.

As showed in **Figure 4**, AI algorithms have been deeply and extensively applied the most in the field of breast cancer. It is perhaps because of the various imaging methods for breast cancer. AI is widely used in mammography (31, 32), magnetic resonance imaging (33, 34), ultrasound (35, 36), and histopathology (37) for tumor classification and segmentation. For instance, Tanaka H. et al. integrated two CNN models and trained them in ultrasound image to achieve a high diagnostic capacity with area under curve (AUC) of 95% (38). Moon et al. reported that their algorithm for detection and classification achieved an AUC of 97% in ultrasound (39). In Mammograms, Mendel et al. used a deep learning algorithm trained on the ImageNet dataset as a feature extractor for malignant and benign distinction and obtained an AUC of 98% on the digital mammography images (40). AI has been successfully used to automatically analyze various breast screening images to improve the diagnostic accuracy of breast cancer.

Overlapping analysis showed that, currently, AI is mostly applied in tumor diagnosis through processing images such as CT, x-ray, B-ultrasound, or endoscopy compared with other applications. With the development of laparoscopic surgery and robotic surgery, AI has also begun to play an important role in the surgical field. Lgaki et al. established a total mesorectal excision plane image-guided navigation system to aid surgeons in recognizing the total mesorectal excision plane for dissection (41). In the field of translational medicine research, AI has also made contributions. Several teams have attempted to use machine learning methods to detect gene mutations in thousands of genes in Next Generation Sequencing (42, 43). Yamashita et al. developed a deep learning model for automated microsatellite instability prediction (44). We believe that, in the near future, AI models may be able to evaluate personalized cancer risk and recommend individual intervention and health management.

There are several limitations in our study. First, despite our best efforts to search the literature, it is not possible to obtain all of them, which may lead to bias. Second, non-English literatures were not included in the analysis. Third, our analysis was based on machine algorithms, which may induce some unavoidable problems, such as repeated analysis, phrases with the same meaning divided into two keywords, and so forth.

## 5 Conclusions

For early tumor detection, precision diagnosis and treatment, drug discovery, and so on, the research on AI in cancer is developing rapidly currently. We summarized indexes of publication and citation including annual growth, most productive countries/regions, most contributing authors, journals, and institutions, co-occurrence keywords, and frontier hotspots in this field. The USA and China are the most major producing countries. American, British, and Japanese authors had the high-quality papers. With the development of deep learning algorithms, significant progress has been made in imaging-assisted diagnosis of many cancer types especially breast cancer and lung cancer. At present, the accuracy of most AI is not satisfactory and can only be applied to some common diseases, mainly due to insufficient available data. AI also faces challenges in terms of data quality, security, and privacy. Despite this, we look forward to the important progress of AI in identifying early cancers, inferring specific cancer sites, personalizing treatment plans, characterizing tumor microenvironment, and predicting prognosis in the future.

## Data availability statement

The raw data supporting the conclusions of this article will be made available by the authors, without undue reservation.

## Author contributions

S-HW and GC contributed equally to this work. S-HW, GC, and XF conceived the project and wrote the manuscript. XZ, TL, and YS collected and collated the data from Web of Science Core Collection. S-HW, GC, and LC accomplished visualization of the data. XF and LC supervised research. LC acquired financial support. All authors discussed the results and comments on the manuscript. All authors contributed to the article and approved the submitted version.

## Supplementary material

Supplementary Figure 1 Word cloud analyses of publication and citation for journals. **(A)** Publication. **(B)** Citation.

Supplementary Figure 2 Exploration of research focus. **(A)** Analysis of keyword density. **(B)** Cluster analysis of keywords.

Sheet 1 Searching strategy.

Sheet 2 The collated documents from Web of Science core collection database.
