# Supplementary figures and images for: Global development of artificial intelligence in cancer field: a bibliometric analysis range from 1983 to 2022
Source: Front Oncol. 2023 Jul 14;13:1215729. doi: 10.3389/fonc.2023.1215729 (PMC10382324; doi:10.3389/fonc.2023.1215729)

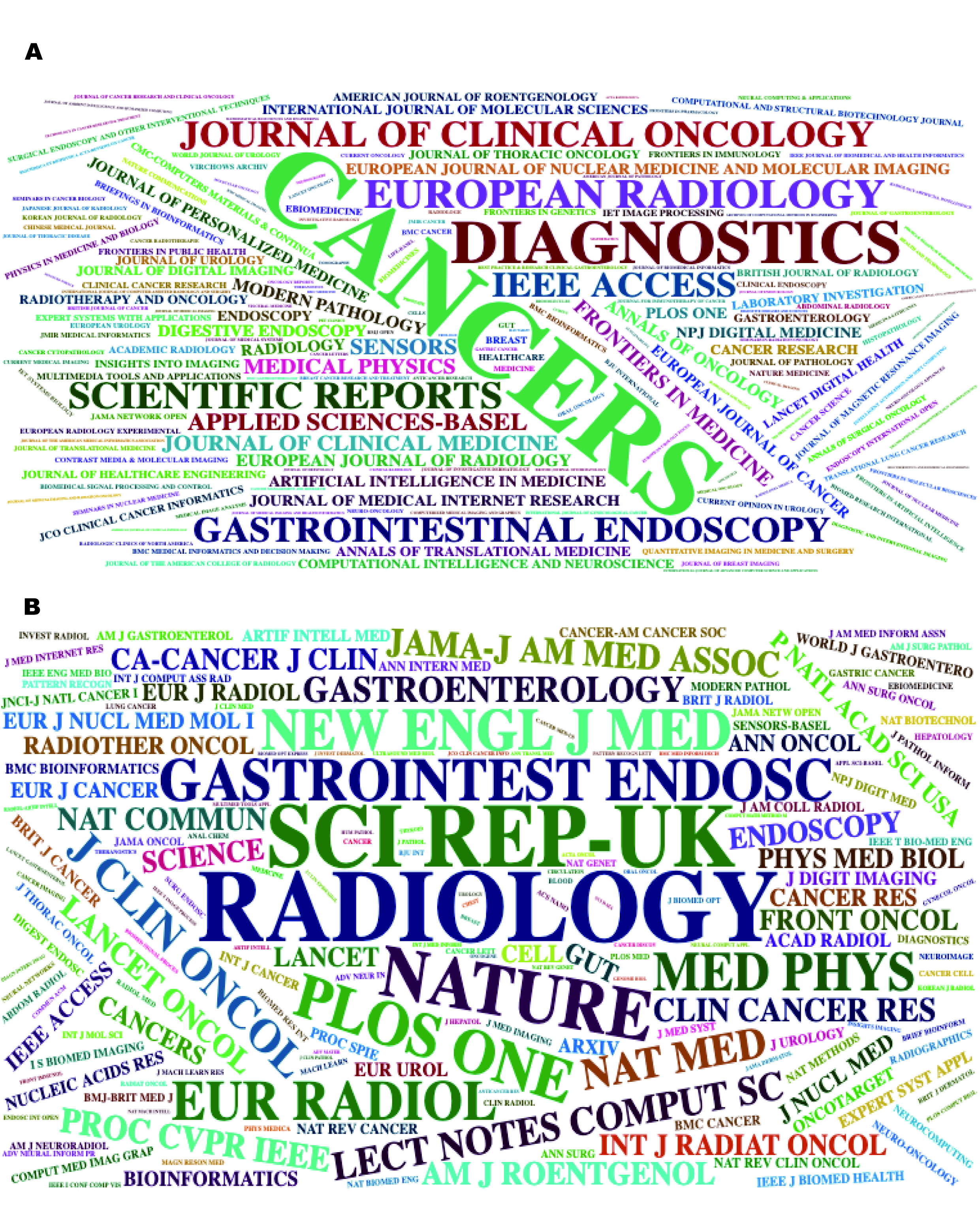

Supplement: Supplementary Figure 1 — Word cloud analyses of publication and citation for journals. (A) Publication. (B) Citation. [file Image_1.tif]

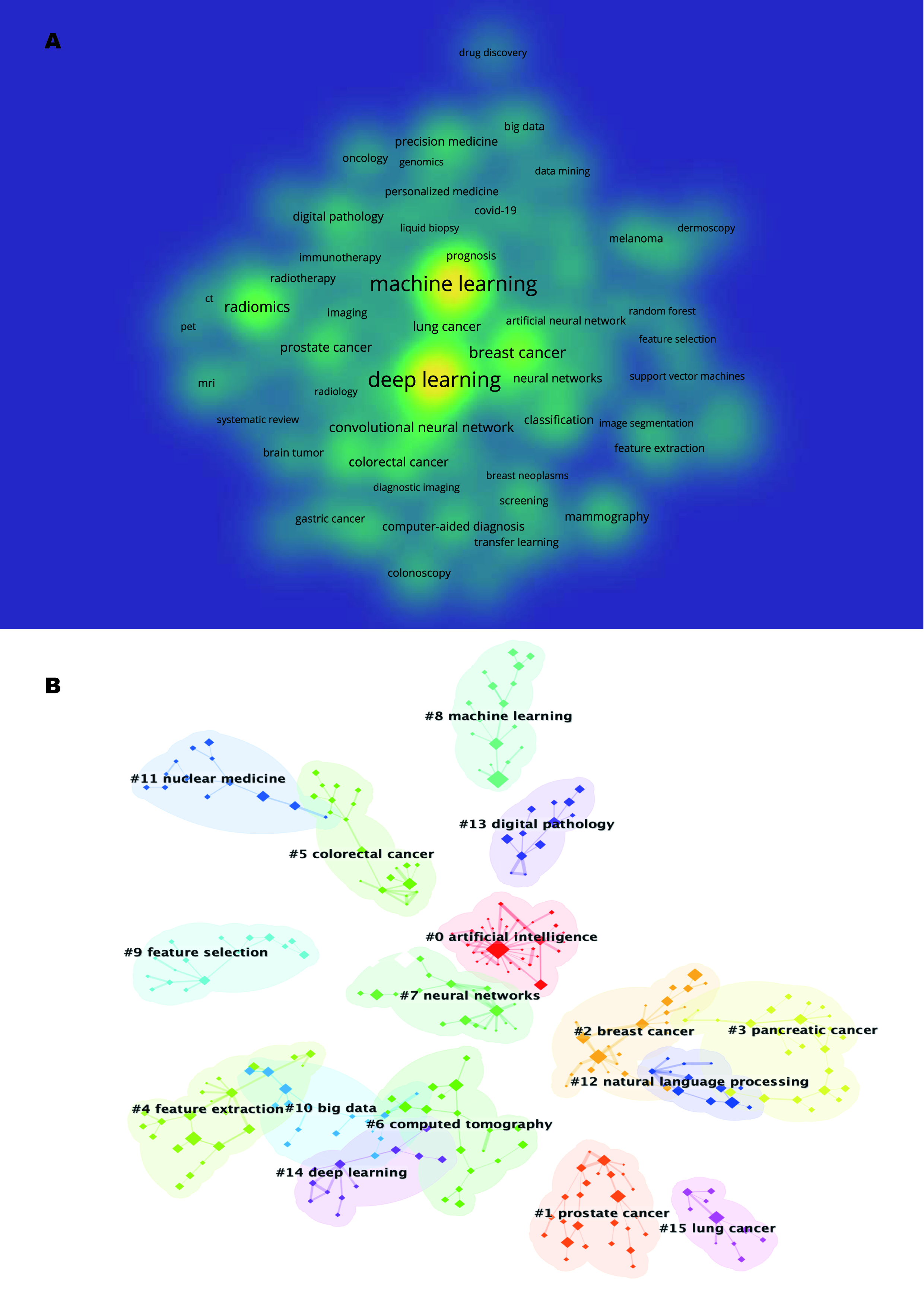

Supplement: Supplementary Figure 2 — Exploration of research focus. (A) Analysis of keyword density. (B) Cluster analysis of keywords. [file Image_2.tif]
